# The Role of Anti-Inflammatory Nutrition in Metabolic Syndrome and Cardiometabolic Diseases

**DOI:** 10.3390/nu18162732

**Published:** 2026-08-21

**Authors:** Cristina-Sorina Cătană, Monica Mihaela Marta, Denisa Popa, Eleonora Dronca

**Affiliations:** 1 Department of Basic Sciences, Iuliu Hațieganu University of Medicine and Pharmacy, 8 Victor Babes, Street, 400012 Cluj-Napoca, Romania; ccatana0128@gmail.com; 2 Department of Medical Education, Iuliu Hațieganu University of Medicine and Pharmacy, 8 Victor Babes, Street, 400012 Cluj-Napoca, Romania; 3 Department of Laboratory Medicine, Transilvania Hospital, 1-3 René Descartes Street, 400486 Cluj-Napoca, Romania; popa.deni7@yahoo.com; 4 Department of Medical Genetics, Iuliu Hațieganu University of Medicine and Pharmacy, 8 Victor Babes, Street, 400012 Cluj-Napoca, Romania; eleonora.dronca@umfcluj.ro

**Keywords:** anti-inflammatory nutrition, metabolic syndrome, cardiometabolic disease, plant-based diet, gut microbiota, chronic inflammation, endothelial dysfunction, microbiota-derived metabolites, immunomodulation

## Abstract

**Background**: Metabolic syndrome is closely linked to chronic systemic inflammation and intestinal dysbiosis. Anti-inflammatory nutrition is an approach based on the interaction between dietary intake, chronic low-grade inflammation, and the pathophysiological mechanisms of metabolic syndrome and cardiometabolic diseases. This narrative review evaluates the impact of plant-based dietary models, including vegan and modified Mediterranean variants, on key metabolic and inflammatory parameters. **Methods**: Literature data published in PubMed, Science Direct, Scopus, and Google Scholar between 2017 and 2026 were used to evaluate the relation between diet, inflammation, and cardiometabolic diseases. We focused on multiple biomarkers, including inflammatory, metabolic, and oxidative-stress indicators measured by standardized laboratory methods to estimate the overall relationship between diet and chronic inflammation. **Results**: Dietary indices such as eADI-17 allow quantification of the inflammatory potential of anti-inflammatory nutrition and correlate with biomarkers such as hsCRP, IL-6, and soluble TNF receptors. **Conclusions**: Integrated nutritional strategies that combine plant-based models, temporal control of meals, and microbiota-directed interventions provide efficient support by improving endothelial dysfunction, the thrombotic profile, and chronic inflammation.

## 1. Introduction

### 1.1. Theoretical Foundations and Pathophysiological Mechanisms of Chronic Low-Grade Inflammation in Metabolic Diseases

The concept of anti-inflammatory nutrition is based on the close interaction between dietary intake, chronic low-grade inflammation, and the pathophysiological mechanisms of metabolic syndrome and cardiometabolic diseases. Chronic low-grade inflammation is driven by exogenous nutrient overload, macrophage polarization, and Toll-like receptor activation, which are critical sentinel proteins of the innate immune system involved in inflammatory pathways interfering with insulin signaling [1]. Metabolic syndrome is defined as a group of conditions that include central obesity, dyslipidemia, hypertension, and insulin resistance, being closely linked to systemic inflammation and intestinal dysbiosis. Hyperglycemia in the context of insulin resistance, the release of pro-inflammatory cytokines from adipose tissue, and oxidative stress contribute to endothelial dysfunction, thus worsening hypertension and accelerating atherosclerosis [2]. In addition, metabolic syndrome is associated with a prothrombotic state characterized by the activation of coagulation factors and reduced fibrinolysis, which amplifies the risk of arterial and venous thrombotic events [3]. In this context, dietary models based on plant foods have been extensively investigated as nutritional strategies with anti-inflammatory and cardioprotective potential [4]. Plant-based diets, which include both vegetarian and vegan models, are characterized by a lower frequency of consumption of foods of animal origin, a high fiber content, and a low intake of saturated fatty acids. These characteristics correlate with improved insulin sensitivity, better glycemic control, and a lower risk of type 2 diabetes, as well as favorable lipid profiles, with reduced LDL cholesterol and triglycerides [5]. Plant-based omega-3 fatty acids and phytonutrients with antioxidant and anti-inflammatory properties further contribute to reducing C-reactive protein and other inflammatory markers [6]. The Mediterranean diet and its variants enriched with bioactive compounds illustrate a central model of anti-inflammatory nutrition. A modified, “green” version of the Mediterranean diet, enriched with polyphenols from Mankai, walnuts, and green tea, reduced intrahepatic fat, diastolic blood pressure, LDL cholesterol, and C-reactive protein in individuals with abdominal obesity or dyslipidemia [7]. The bioactive components of this model include monounsaturated fatty acids from extra-virgin olive oil, omega-3 polyunsaturated fatty acids, fiber, dietary nitrates, and diverse polyphenols, which attenuate oxidative stress by scavenging free radicals, inhibit NF-κB-dependent inflammatory cascades, and increase nitric oxide bioavailability by stimulating eNOS expression and preventing eNOS uncoupling [5,8]. Strictly vegan diets provide a dietary framework that is very low in cholesterol and saturated fats, but rich in fiber, vitamins, antioxidants, and complex carbohydrates. Adherence to a well-structured vegan diet is associated with a lower body mass index and waist circumference compared with omnivores, reduced energy density, improved weight control, and improved insulin sensitivity through the effect of soluble fiber on postprandial glycemia [9]. By completely excluding saturated fats, the vegan diet favors a lipid profile characterized by lower LDL cholesterol and triglyceride values, while its high potassium, magnesium, and polyphenol content contributes to lower blood pressure through vasodilation and antioxidant effects. Polyphenols are a defining element of anti-inflammatory nutrition [10]. In vegetarian and vegan diets, polyphenols from vegetables, fruits, spices, and green tea counteract oxidative stress and inflammation, and they may increase insulin sensitivity, improve lipid metabolism, and reduce blood pressure. In particular, olive oil polyphenols reduce visceral adiposity, insulin resistance, blood pressure, and lipid peroxidation, while simultaneously inhibiting NF-κB signaling and the production of pro-inflammatory cytokines, with favorable effects on metabolic health [11]. Compounds such as epigallocatechin gallate from green tea and curcumin exert epigenetic effects that repress the expression of the inflammatory genes involved in the pathophysiology of metabolic syndrome [12].

Chronic low-grade inflammation is a central feature of metabolic diseases, especially metabolic syndrome, where disturbances of glucose metabolism, endothelial dysfunction, and the prothrombotic state converge through common inflammatory pathways [13].

The relationship between nutrition and inflammation is fundamentally mediated by the intestinal microbiota. The increased intake of fiber, polyphenols, and resistant starch in vegan diets shapes the composition of microbiota, increasing the abundance of species capable of fermenting complex polysaccharides and producing short-chain fatty acids such as butyrate. These metabolites have systemic anti-inflammatory effects, so they improve intestinal barrier function and modulate glucose and lipid metabolism [14,15]. At the same time, plant-based diets reduce circulating trimethylamine N-oxide (TMAO) levels and decrease the abundance of endotoxin-producing bacteria, such as *Bilophila wadsworthia* and *Escherichia coli*, whose lipopolysaccharides promote metabolic endotoxemia and chronic low-grade inflammation, recognized as a driver of insulin resistance and atherogenesis [6].

Persistent inflammation correlates with activation of oxidative stress pathways, which contributes to endothelial dysfunction, worsening hypertension, and promotion of atherosclerosis [1,2]. This pathogenic network places metabolic syndrome at the center of cardiometabolic diseases, in which inflammation, coagulation, and endothelial injury potentiate one another. The increasing global prevalence of metabolic syndrome in developed as well as in low- and middle-income countries is linked to urbanization, reduced physical activity, and diets rich in ultra-processed foods. Hyperglycemia in the context of insulin resistance is associated with disruption of the PI3K/AKT pathway and overactivation of the MAPK pathway. Dysregulation of PI3K/AKT reduces GLUT-4 translocation, decreases eNOS expression and nitric oxide production, thereby compromising vasodilation [16]. In parallel, MAPK overactivation, stimulated by hyperinsulinemia, TNF-α, and resistin, intensifies the expression of inflammatory genes and adhesion molecules mediated by NF-κB, thus promoting oxidative stress and endothelial dysfunction [17]. Obesity, a major component of metabolic syndrome, is closely linked to chronic low-grade inflammation through remodeling of the immune compartment of adipose tissue. Expansion of adipose mass leads to increased recruitment of monocytes and their differentiation into M1 macrophages, which have a pro-inflammatory phenotype. Resident macrophages in adipose tissue increase from approximately 4% of immune cells in normal-weight individuals to about 12% in obesity, their accumulation being more pronounced in visceral than in subcutaneous adipose tissue. M1 macrophages produce increased levels of pro-inflammatory cytokines, such as IL-6 and TNF-α, which induce chronic inflammation, recruit additional inflammatory cells, and worsen insulin resistance. MCP-1 produced by preadipocytes amplifies monocyte recruitment into adipose tissue, where they transform into TNFα-secreting macrophages involved in obesity-associated insulin resistance [18]. Remodeling of the lymphocyte repertoire in adipose tissue further contributes to low-grade inflammation [19]. In obesity, immune cells adopt a pro-inflammatory profile, with accumulation of cytotoxic CD8+ T cells and Th1 cells, which promote insulin resistance, whereas the proportion of regulatory T cells (Treg) and Th2 cells, which are protective against insulin resistance, decreases significantly [20]. In lean mice, Treg cells account for approximately 50% of the CD4+ T-cell compartment in adipose tissue, a percentage that decreases substantially in obesity, favoring loss of immunological tolerance and perpetuation of chronic inflammation. In addition to local effects, adipocytes and immune cells in adipose tissue secrete TNF-α and other key cytokines that alter lipid metabolism and sustain metabolic complications. Adipokines represent another link between excess adiposity and systemic inflammation.

Expansion of adipose tissue leads to the release of leptin, TNF-α, adiponectin, and IL-6 [21]. Circulating IL-6 stimulates the hepatic production of acute-phase proteins, including C-reactive protein, serum amyloid A, fibrinogen, haptoglobin, and alpha-1- antichymotrypsin, thereby amplifying the inflammatory response and favoring a prothrombotic state [22]. IL-6 also induces hepcidin and megakaryocyte maturation in the bone marrow, with increased platelet secretion, which may contribute to enhanced platelet aggregation. In the presence of TGF-β, IL-6 promotes the differentiation of T helper 17 cells and inhibits Treg formation; the imbalance between Th17 and Treg compromises immune tolerance and supports chronic inflammation. The central role of TNF-α in metabolic disease-related inflammation is supported by clinical and mechanistic studies [23]. In patients with type 2 diabetes, serum TNF-α levels are significantly higher than in healthy subjects, while increased values correlate positively with glycated hemoglobin, suggesting involvement in the pathogenesis of diabetes through interference with peripheral insulin action. In obesity, increased TNF-α and free fatty acids lead to chronic activation of c-Jun N-terminal kinase (JNK) in the liver, muscle, and adipose tissue, disrupting insulin signaling through serine phosphorylation of the Insulin Receptor Substrate (IRS-1) [24]. The binding of TNF-α to the TNFR1 receptor activates IKK, releasing NF-κB and increasing transcription of inflammatory genes. TNF-α also stimulates sphingomyelinase, increases ceramide levels, and activates PP2A, which dephosphorylates and inactivates AKT, a critical mediator of glucose uptake and glycogen synthesis. These cascades converge toward insulin resistance and exacerbate inflammation. In the context of metabolic syndrome, chronic low-grade inflammation is inseparable from oxidative stress and endothelial dysfunction [13,24,25]. The low-grade inflammation generated by the components of metabolic syndrome worsens vascular dysfunction and metabolic disturbances, including insulin resistance and hepatic steatosis, which justifies therapeutic approaches that simultaneously target metabolic control and reduction in inflammation [26].

In this context, metabolic syndrome is not merely a statistical aggregation of risk factors, but a pathophysiological construct that integrates metabolic disturbances, inflammation, and thrombosis, with major effects on cardiovascular (CVD) mortality and morbidity. Systemic and local inflammation accelerates the formation and instability of atherosclerotic plaques, increasing the likelihood of acute events such as myocardial infarction and stroke. Together, these mechanisms describe a cardiometabolic spectrum in which metabolic disturbances converge toward thrombosis and extensive vascular injury. Visceral obesity appears as a nodal element in this spectrum.

A dietary intervention could target amino acid metabolism. For example, L-arginine supplementation, which acts as a substrate for cellular NO and is obtained from plant-based diets (nuts and seeds) or mixed and Western diets (fish, grains), can have cardioprotective effects. However, certain amino acids, notably methionine and branched-chain amino acids (BCAAs) such as leucine, isoleucine, and valine, have an adverse impact on CVD risks [27]. High blood levels of these amino acids have pro-inflammatory effects, inducing insulin resistance or atherosclerosis, while glycine and L-glutamine are beneficial by supporting vascular function, reducing oxidative stress, and improving metabolic health [28]. Therefore, while current research has contributed to a better understanding of CVD, significant understanding gaps remain, especially regarding rare amino acids and their connection with metabolic components [29].

### 1.2. Inflammatory, Metabolic and Thrombotic Biomarkers

Profiling inflammatory, metabolic, and thrombotic biomarkers clarifies how metabolic syndrome correlates with chronic low-grade inflammation and hypercoagulability. Obesity is associated with the increased secretion of pro-inflammatory cytokines, especially IL-6 and TNF-α, and with the accumulation of macrophages in adipose tissue. This chronic inflammation increases the expression of plasminogen activator inhibitor-1 (PAI-1) and intensifies platelet aggregation, impairing fibrinolysis and favoring thrombosis. Hypertension contributes to endothelial activation and oxidative stress, which stimulate pro-inflammatory pathways such as NF-κB, and is associated with increases in fibrinogen and thrombotic activity. Dyslipidemia, through the oxidation of LDL particles, triggers inflammatory cascades, accelerates atheroma plaque formation, and increases platelet reactivity and coagulation factor levels, amplifying thrombotic risk [1,2]. Insulin resistance, in turn, activates macrophages, reduces endothelial nitric oxide synthesis, increases the production of reactive oxygen species, and intensifies platelet reactivity, leading to hypercoagulability and increased platelet aggregation. In the presence of adipokines and cytokines, the expansion of adipose mass leads to the release of leptin, adiponectin, TNF-α, and IL-6. Leptin, which can act as an inflammatory mediator, is involved in several inflammatory diseases [30]. Chronic inflammation of metabolic or autoimmune origin can induce central leptin resistance, further increasing its circulating levels and worsening inflammation. IL-6 has a central role in the acute phase: it acts on the liver and rapidly induces the synthesis of C-reactive protein, serum amyloid A, fibrinogen, haptoglobin, and alpha-1-antichymotrypsin, marking the interface between inflammation and the prothrombotic state [31]. The same cytokine increases hepcidin and megakaryocyte maturation, with increased platelet secretion, which may contribute to enhanced platelet aggregation. In the presence of TGF-β, IL-6 stimulates the differentiation of T helper 17 cells and inhibits Treg formation; the imbalance between these lymphocyte subsets compromises immune tolerance and supports chronic inflammation. TNF-α appears as a central node in the inflammatory biomarker network of metabolic diseases. A recent study showed that serum TNF-α levels are significantly higher in patients with type 2 diabetes mellitus than in healthy subjects, while increased concentrations correlate positively with glycated hemoglobin, suggesting the involvement of this cytokine in the pathogenesis of diabetes by interfering with peripheral insulin action. In obesity, increased TNF-α and free fatty acids determine the chronic activation of JNK in the liver, muscle, and adipose tissue, disrupting insulin signaling through serine phosphorylation of IRS-1. The binding of TNF-α to the TNFR1 receptor activates IKK, releasing NF-κB and increasing transcription of inflammatory genes. TNF-α also stimulates sphingomyelinase, increases ceramide levels, and activates PP2A, which dephosphorylates and inactivates AKT, which is critical for glucose uptake and glycogen synthesis. In addition, TNF-α increases lipolysis and free fatty acids, which favors DAG accumulation and activation of PKC isoforms with additional serine phosphorylation of IRS-1. The same cytokine increases the expression and activity of PTP1B, the enzyme that dephosphorylates IRS-1 and IRS-2, further impairing insulin signaling. On the thrombotic component, TNF-α intensifies tissue factor expression and promotes thrombotic activity through activation of the NF-κB pathway, providing a direct molecular link between inflammation and coagulation. As far as the thrombotic risk is concerned, several plasma biomarkers reflect the hypercoagulability associated with the components of metabolic syndrome [6,25]. In hypertensive patients, D-dimer levels are higher than in normotensive subjects, with values increasing with the severity of hypertension, which suggests an increased tendency toward clot formation and its role in thromboembolic complications. Plasma fibrinogen is also significantly higher in patients with hypertension, a finding that has been consistently reported in several studies. The concomitant increase in fibrinogen and PAI-1 is highlighted as a major factor in exacerbating thrombotic risk in metabolic syndrome, because these proteins promote coagulation and reduce clot breakdown [32]. The comparative table of metabolic syndrome components emphasizes that obesity increases fibrinogen and coagulation factors and favors platelet aggregation through leptin, whereas hypertension increases fibrinogen, PAI-1, and D-dimer. Dyslipidemia amplifies platelet hyperactivity and increases coagulation factors, while insulin resistance promotes hypercoagulability by increasing fibrinogen and other prothrombotic factors and by altering platelet reactivity. Integrative analysis of these biomarkers shows that metabolic syndrome is simultaneously associated with inflammatory markers (CRP, IL-6, TNF-α), disturbances in insulin signaling, and thrombotic markers (fibrinogen, PAI-1, D-dimer, tissue factor, platelet aggregation), defining a complex pathobiological profile that supports both metabolic progression and the risk of arterial and venous thromboembolism [6]. Data from the EPIC cohort show that certain dietary components containing dicarbonyls were associated with fatal and non-fatal CVD risk even after adjustment for hs-CRP and other risk factors; these observations suggest that dietary intake may influence the balance between inflammation and the tendency toward coagulation [33].

TCA cycle metabolites are also involved in inflammation and thrombosis. Succinate accumulates in excess during inflammation and cellular stress, stabilizing hypoxia-inducible factor 1-alpha (HIF-1α) and turning on pro-inflammatory genes to initiate pro-inflammatory cytokine production (like IL-1β) in macrophages [34]. Citrate, which is upregulated in activated immune cells, moves from mitochondria to cytosol to provide acetyl-CoA for fatty acid synthesis and inflammatory mediator production [35]. Itaconate, synthesized from TCA cis-aconitate, harbors anti-inflammatory and antioxidant properties. Fumarate, another TCA cycle intermediate, also has immunoregulatory properties; its derivative dimethyl fumarate (DMF) is a therapeutic candidate for the treatment of virus-induced hyper-inflammation and oxidative stress. Upregulation of the glycolysis–TCA axis by shifting cellular energy pathways raises reactive oxygen species (ROS) production in red blood cells and endothelial cells, directly promoting deep vein thrombosis (DVT) and chronic low-grade systemic inflammation as well as elevating metabolic disease risk [36].

Glutathione maintains the immune functionality of Th17 cells and facilitates mitochondrial signal transduction and synthesis of IL-22 protein in these cells [37]. In addition, glutamine plays a key role in CD8+ T cell proliferation and IFN-γ secretion, thus modulating cellular immune responses [38]. Imbalances in glutamate metabolism generate multiple pro-inflammatory responses by increasing ROS, which activate the IκB kinase (IKK) complex, phosphorylating and degrading IκBα. This liberates NF-κB, a key pro-inflammatory factor that moves into the cell nucleus to activate inflammation-related genes [39]. Moreover, overactivation of glutamate receptors, such as NMDA receptors, increases intracellular Ca^2+^ levels that activate protein kinase C, which in turn activates the IKK complex, leading to IκBα phosphorylation and activation of the pro-inflammatory NF-κB pathway [39,40]. The glutamine/glutamate ratio represents an important clinical biomarker used in patient diagnosis. On the one hand, a lower ratio estimates a higher risk of coronary artery disease (CAD), which is important for early detection. On the other hand, regarding treatment intervention, glutamine supplementation has cardioprotective effects after myocardial infarction, aiming to improve CVD outcomes.

This narrative review aims to investigate whether chronic low-grade inflammation could be modulated by the nutritional spectrum through dietary components that either suppress or activate immune, metabolic, and thrombotic pathways.

## 2. Search Methodology

For this narrative review on anti-inflammatory diets, metabolic syndrome, and cardiovascular pathology, we conducted a structured search for English-language literature data published in PubMed, Science Direct, Scopus, and Google Scholar between 2017 and 2026.

The search terms were obtained by combining keywords from two distinct groups. One group included “anti-inflammatory nutrition”, “dietary inflammatory index”, “polyphenols”, “Mediterranean diet”, “non-lipid metabolites”, and “amino acid metabolism”, while the other group consisted of “cardiovascular disease”, “metabolic syndrome”, “inflammation”, “endothelial dysfunction”, and “immunomodulation”.

We included randomized controlled trials, case–control studies, prospective studies, comprehensive reviews, systematic reviews and meta-analyses that evaluated anti-inflammatory nutritional patterns alongside low-grade inflammation biomarkers such as CRP, IL-6, and TNF-α and their cardiometabolic endpoints. We excluded all studies focusing on isolated single-nutrient overdoses or lacking clear cardiometabolic results. This search generated a total of 739 records. Publications that did not quantify dietary effects on metabolic health and vascular dysfunction were excluded, thus obtaining 95 records. The titles and abstracts of the identified records were screened. In case of disagreement, the articles were selected by discussion. Next, the full text of the selected records was assessed. Here too, in case of disagreement, the articles were selected by discussion.

A specific search for original studies using the above-mentioned terms generated a total number of 485 clinical studies. After excluding the studies on animals, we obtained 362 human clinical studies. Out of these, we also excluded the studies focusing on other pathologies, thus obtaining 312 records. After excluding isolated single-nutrient overdoses, 58 records were obtained. Forty-one of these did not include inflammatory, vascular, and metabolic marker outcomes, so the remaining 17 high-quality clinical studies were summarized in Table 1, Table 2 and Table 3. Once the relevant literature was selected, we used study design, sample size, dietary intervention, duration, and documented changes in metabolic and inflammatory parameters to identify physiological mechanisms such as oxidative stress reduction, microbiota modulation, and cytokine regulation.

## 3. Anti-Inflammatory and Pro-Inflammatory Dietary Models

### 3.1. Dietary Models with Anti-Inflammatory Potential

**Figure 1:**
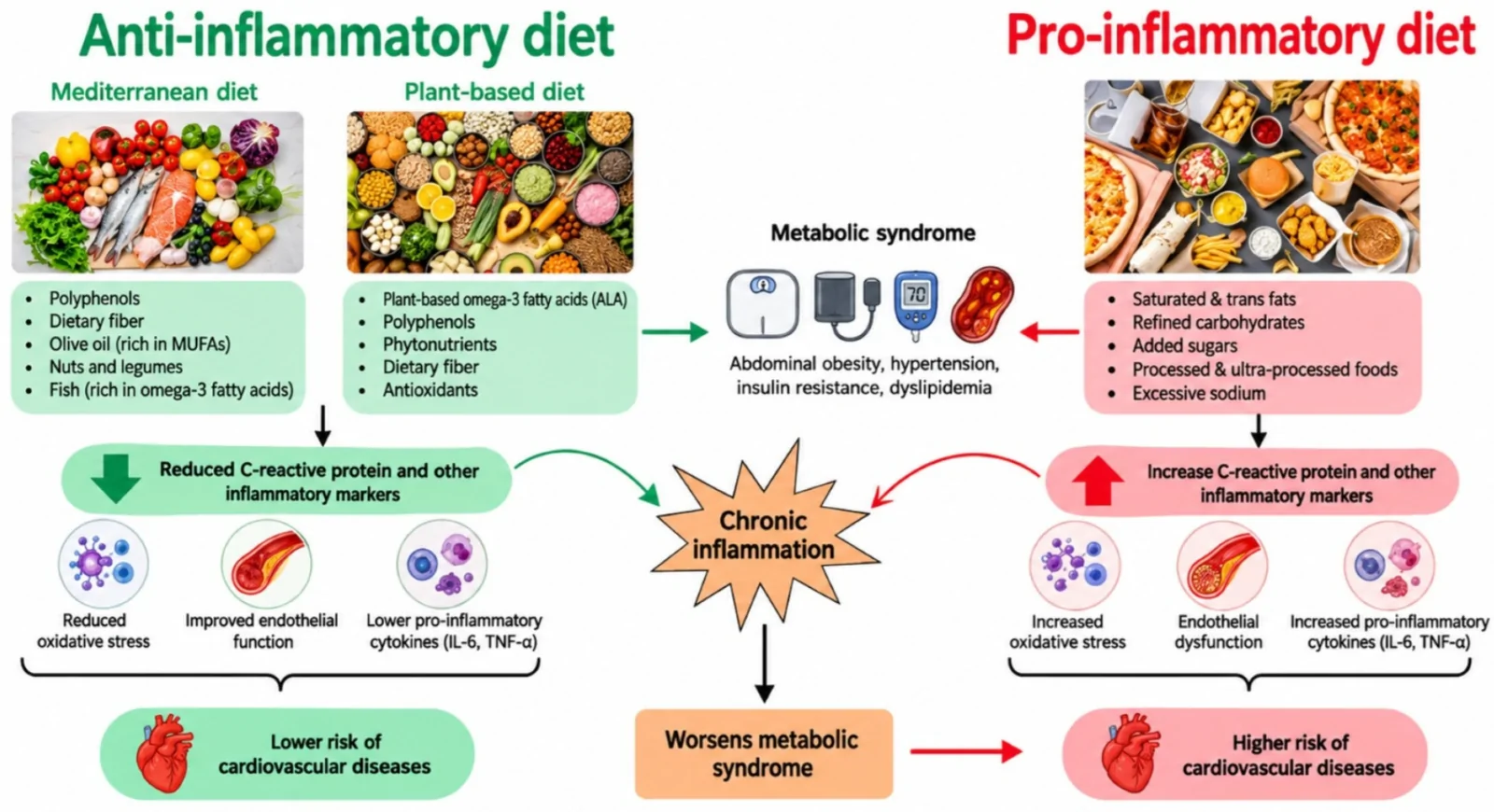
Chronic inflammation modulated by the nutritional spectrum. Green arrows indicate the beneficial effects associated with anti-inflammatory dietary patterns. Red arrows point to the detrimental effects associated with pro-inflammatory dietary patterns. **Abbreviations**: MUFA: monounsaturated fatty acids; ALA: alpha-linolenic acid.

Plant-based dietary models are characterized by a lower frequency of consumption of foods of animal origin, increased fiber intake, and low saturated fat content, and are described as patterns with highly favorable effects on metabolic and vascular health. Transitioning eating habits from pro- to anti-inflammatory diets reduces oxidative stress and systemic inflammatory biomarkers and improves endothelial dysfunction, as shown in Figure 1.

Anti-inflammatory models include vegetarian and vegan diets rich in phytonutrients, polyphenols, and unsaturated fatty acids, which are associated with improved insulin sensitivity, glycemic control, lipid profiles, and reduced inflammatory markers such as C-reactive protein (CRP) and interleukin-6 (IL-6). Plant-based diets also provide omega-3 fatty acids of plant origin and other bioactive compounds with antioxidant and anti-inflammatory effects. These compounds contribute to lowering oxidative stress and attenuating pro-inflammatory pathways, including those mediated by nuclear factor kappa B (NF-κB), with profound implications for the risk of atherosclerosis and thrombosis [6]. The clinical and epidemiological relevance of anti-inflammatory models is highlighted by large-scale observational data, such as national analyses based on the National Health and Nutrition Examination Survey (NHANES) cohort. These evaluations have established that a pro-inflammatory dietary index is strongly associated with elevated all-cause mortality and cardiovascular disease-related mortality in individuals with metabolic syndrome, thus strengthening the link between dietary patterns and long-term cardiometabolic risk [41]. These findings suggest that reducing the inflammatory load of the diet represents a pragmatic, highly effective direction in the management strategy for metabolic syndrome, although long-term randomized interventions are necessary to confirm clinical effects and define the underlying molecular mechanisms.

The vegan diet, as an extreme form of plant-based nutrition, fully eliminates animal products and has a low caloric density and very low cholesterol and saturated fat content, but a high intake of fiber, complex carbohydrates, vitamins, antioxidants, and minerals. A central advantage of this model is its association with lower body mass index (BMI) and waist circumference compared with omnivores, reflecting a more favorable body composition and reduced visceral obesity. Increasing fiber intake, especially soluble fiber, is essential for obesity control by promoting satiety, reducing energy intake, and improving postprandial glycemia, thus improving insulin sensitivity. A vegan diet may also increase the expression of adiponectin, an adipokine with anti-inflammatory and insulin-sensitizing properties that is often reduced in individuals with metabolic syndrome. The absence of saturated fats and the increased intake of monounsaturated and polyunsaturated fatty acids translate into lower low-density lipoprotein cholesterol (LDL-C) and triglyceride levels, establishing a lipid profile highly favorable for the prevention of atherosclerosis. This dietary model is also associated with reductions in systolic and diastolic blood pressure, an effect attributed in part to its high potassium and magnesium content, which support vasodilation and reduce peripheral vascular resistance [42].

To validate these physiological pathways, several key randomized clinical trials have evaluated the metabolic and cardiometabolic impact of vegan and whole-food plant-based (WFPB) dietary patterns. Table 1 presents the main findings of such recent clinical trials.

**Table 1:** Cardiorenal and metabolic effects of dietary interventions.

Study Design	Population	Intervention	Duration	Main Findings	References
Randomized crossover clinical trial with weekly cooking classes	40 adults with a ≥5% cardiovascular disease risk	WFPB vegan diet with high EVOO (4 tablespoons/day) vs. WFPB vegan diet with low EVOO (<1 teaspoon/day) separated by a 1-week washout	8 weeks total (4 weeks per phase)	Both diets reduced LDL-C, apoB, glucose, and hs-CRP; the low-EVOO diet produced a greater LDL-C reduction.	[43]
Randomized, parallel, identical-twin trial	22 pairs (44 healthy adult identical twins)	Healthy vegan diet vs. healthy omnivorous diet, with meals provided for the first 4 weeks	8 weeks	Significant reductions in LDL-C, fasting insulin, and body weight in vegan twins.	[44]
Parallel, open-label randomized controlled trial	244 overweight or obese adults	Low-fat vegan diet vs. control diet (no dietary changes)	16 weeks	Vegan diet significantly reduced potential renal acid load and net endogenous acid production; direct correlation with visceral fat loss.	[45]
Parallel, open-label randomized controlled trial	75 overweight or obese adults with no history of diabetes	Low-fat vegan diet vs. control diet (no dietary changes)	16 weeks	Saturated and trans fat restriction + increased relative linoleic and alpha-linolenic acid intakes decreased fat mass and HOMA-IR, and enhanced insulin secretion.	[46]
Parallel, open-label randomized controlled trial	75 overweight or obese adults with no history of diabetes	Low-fat vegan diet vs. control diet (no dietary changes)	16 weeks	Vegan diet better reduced body weight, fat mass, and HOMA-IR; reduced leucine and histidine intakes correlated with lower fat mass and insulin resistance.	[47]
Randomized parallel controlled trial	65 overweight or obese adults with ≥ comorbidity (type 2 diabetes, ischemic heart disease, hypertension, hypercholesterolemia)	Non-energy-restricted WFPB diet with twice-weekly educational meetings vs. normal care; WFPB supplemented with vitamin B12	12 weeks (with follow-up to 6 and 12 months)	Significant BMI reduction in the WFPB group at 6 and 12 months; total cholesterol reduction at 12 months.	[48]

**Abbreviations:** apoB, Apolipoprotein B; BMI, Body mass index; CVD, Cardiovascular disease; EVOO, Extra virgin olive oil; HOMA-IR, Homeostatic model assessment of insulin resistance; hs-CRP, High-sensitivity C-reactive protein; LDL-C, Low-density lipoprotein cholesterol; NEAP, Net endogenous acid production; PRAL, Potential renal acid load; RCT, Randomized controlled trial; RFHH, Recipe for heart health; T2DM, Type 2 diabetes mellitus; WFPB, Whole-food plant-based (diet).

Dietary polyphenols are a key component of the anti-inflammatory potential of plant-based models. In particular, polyphenols from extra virgin olive oil (EVOO) contribute to reducing visceral adiposity, insulin resistance, blood pressure, and lipid peroxidation, while simultaneously inhibiting NF-κB signaling and decreasing the production of pro-inflammatory cytokines, with metabolic and cardiovascular benefits [49]. Polyphenolic compounds from citrus fruits can block NF-κB expression, reducing oxidative stress and inflammation, which is associated with increased insulin sensitivity, improved lipid metabolism, and lower blood pressure. In addition, phytonutrients such as epigallocatechin gallate (EGCG) from green tea and curcumin from turmeric display epigenetic effects that repress the expression of inflammatory genes involved in the pathophysiology of metabolic syndrome. These actions contribute to increasing HDL-C, reducing LDL-C, and improving body mass index and blood pressure, together with improved endothelial function and reduced oxidative stress [6].

The Mediterranean diet, especially the “green” version enriched with polyphenols from Mankai (*Wolffia globosa*), walnuts, and green tea, provides a well-characterized anti-inflammatory and cardioprotective model. In individuals with abdominal obesity or dyslipidemia, this modified model reduced intrahepatic fat, diastolic blood pressure, LDL cholesterol, and C-reactive protein, suggesting an improved inflammatory and metabolic status. The bioactive components of the Mediterranean diet include monounsaturated fatty acids from extra-virgin olive oil, omega-3 polyunsaturated fatty acids, fiber, dietary nitrates, and diverse polyphenols. These components attenuate oxidative stress by scavenging free radicals, suppress NF-κB-dependent inflammatory cascades, and increase nitric oxide bioavailability by stimulating eNOS expression and preventing eNOS uncoupling, thereby improving endothelial function and reducing cardiometabolic risk [50]. The beneficial effects of plant-based diets were also documented in neuroinflammation and metabolic pathology, as summarized in Table 2.

**Table 2:** Positive effects of plant-based diet on neuroinflammation and metabolic function.

Study Design	Population	Intervention	Duration	Main Findings	References
Multicenter, randomized, parallel-group, lifestyle intervention trial	6874 (or sub-cohorts) elderly adults (55–75 years) with metabolic syndrome	Intensive weight loss with erMedDiet and physical activity promotion vs. control MedDiet	8 years (longitudinal analysis)	erMedDiet + physical activity reduced the risk of eGFR decline to <60 mL/min/1.73 m^2^ by 40%.	[51]
Randomized, parallel-group, single-blind clinical trial	294 adults with abdominal obesity and dyslipidemia (mean BMI: 31.3)	HDG vs. MED (+28 g/day walnuts) vs. green-MED (walnuts + 3–4 cups/day green tea + 100 g Mankai shake daily); restricted red/processed meat)	18 months	Double proportional IHF% loss in Green-MED group; NAFLD reduced by half.	[52]
Randomized, parallel-group, single-blind clinical trial (MRI substudy)	294 adults with abdominal obesity and dyslipidemia	HDG vs. standard MED vs. Green-MED, all accompanied by physical activity guidance and gym memberships	18 months	Reduced visceral fat in Green-MED group.	[53]
Randomized, parallel-group, single-blind clinical trial (Brain MRI substudy)	284 adults with abdominal obesity and dyslipidemia	HDG vs. standard MED vs. Green-MED, tracking structural changes via automated NeuroQuant MRI segmentation	18 months	Age-related brain atrophy attenuated by 50% in MED and Green-MED groups; brain protection directly mediated by HbA1c reduction and polyphenol absorption.	[54]

**Abbreviations:** BMI, Body mass index; CI, Confidence interval; eGFR, Estimated glomerular filtration rate; er-MedDiet, Energy-reduced Mediterranean diet; Green-MED, Green Mediterranean diet; HbA1c, Glycated hemoglobin; HDG, Healthy dietary guidelines; HR, Hazard ratio; IHF, Intrahepatic fat; MED, Mediterranean diet; MRI, Magnetic resonance imaging; NAFLD, Non-alcoholic fatty liver disease.

Vascular endothelial function is a primary clinical target in the management of cardiometabolic diseases, as endothelial damage mediated by ROS drives atherosclerotic plaque progression. A randomized controlled study in patients with peripheral arterial disease and hypercholesterolemia evaluated a plant-based dietary counseling program compared with no specific dietary intervention. After four months, the group following the plant-based diet showed significant improvements in vascular endothelial function compared with the control group, including a 7.6% reduction in total cholesterol, a 13.6% reduction in LDL, and improvement in flow-mediated dilation, associated with reduced pulse wave velocity. These changes suggest increased nitric oxide production and reduced arterial stiffness, mechanisms through which a plant-based diet may diminish vascular inflammation and the risk of cardiovascular events [55]. Data from another controlled clinical study indicate that a plant-based regimen rich in fiber and complex carbohydrates may reduce the serum levels of IL-6, IL-8, and TNF-α, simultaneously maintaining bone health and favorable changes in body composition, although some molecular markers (e.g., DKK1 or Wnt expression) may fluctuate and should be interpreted carefully [56]. Table 3 summarizes relevant studies on the cardiometabolic effects of various diets.

**Table 3:** Cardiometabolic effects of vegan and vegetarian diets compared to other cardioprotective diets.

Study Design	Population	Intervention	Duration	Main Findings	References
Open-label, randomized parallel trial with a blinded endpoint	100 patients with angiographically defined CAD (≥50% stenosis) without prior MI or bypass surgery in prior 3 months	Vegan diet vs. AHA-recommended diet; groceries and educational materials provided weekly to both groups	8 weeks	hs-CRP level significantly decreased by vegan diet; no significant differences in weight, glycemic parameters, or general lipids.	[57]
Randomized, open-label, crossover dietary trial	118 overweight omnivorous adults with a low-to-moderate cardiovascular risk profile	Low-calorie lacto-ovo VD vs. low-calorie MD, separated by no washout	6 months total (3 months per phase)	Both diets reduced body weight and fat mass; VD reduced LDL-C; MD lowered triglycerides and interleukin-17.	[58]
Randomized, open-label, crossover trial (subgroup analysis)	107 subjects with medium-to-low cardiovascular risk	Low-calorie lacto-ovo VD vs. low-calorie MD	6 months total (3 months per phase)	Vegetarian phase significantly improved creatinine, BUN, and eGFR; no changes observed during the MD phase.	[58]
Randomized, open-label, crossover trial (subgroup analysis)	54 healthy omnivorous adults	3-month lacto-ovo VD phase	3 months	Vegetarian diet significantly reduced dietary B12 intake and circulating B12 levels, indicating a high risk of deficiency without supplementation.	[58,59]
Randomized crossover clinical trial	31 patients with ischemic heart disease on optimal medical therapy	Isocaloric ready-made VD vs. isocaloric ready-made meat diet, separated by a 4-week washout	8 weeks total (4 weeks per phase)	Vegetarian diet significantly reduced oxidized LDL-C, total cholesterol, and LDL-C.	[60]
Randomized crossover clinical trial	36 generally healthy adults	2 servings/day of PBMA vs. animal meat; rest of diet self-selected	16 weeks total (8 weeks per phase)	PBMA diet significantly reduced LDL-C, body weight, and fasting concentrations of TMAO.	[61]
Randomized crossover clinical trial	40 adults (33 completed) with prediabetes or type 2 diabetes	WFKD vs. Med-Plus diet, separated by no washout	24 weeks total (12 weeks per phase)	No differences in HbA1c; WFKD reduced triglycerides more but significantly elevated LDL-C; WFKD compromised fiber and nutrient intake.	[62]
Controlled crossover trial	14 healthy, physically active young men	Traditional MD for 3 weeks vs. vegan MD for 4 weeks, separated by a 7-day washout	7 weeks total	Vegan MD significantly reduced total cholesterol, LDL-C, SBP, DBP, and neutrophil count.	[63]

**Abbreviations:** AHA, American heart association; BUN, Blood urea nitrogen; CAD, Coronary artery disease; DBP, Diastolic blood pressure; eGFR, Estimated glomerular filtration rate; HbA1c, Glycated hemoglobin; hs-CRP, High-sensitivity C-reactive protein; LDL-C, Low-density lipoprotein cholesterol; MD, Mediterranean diet; Med-Plus, Mediterranean-plus; MI, Myocardial infarction; PBMA, Plant-based meat alternatives; SBP, Systolic blood pressure; TMAO, Trimethylamine N-oxide; VD, Vegetarian diet; WFKD, Well-formulated ketogenic diet.

Vegan diets rich in fiber, polyphenols, and resistant starch are associated with a microbial profile distinct from that of omnivorous diets. Vegan individuals show increased abundance of *Prevotella* species, specialized in fermenting complex polysaccharides, and a lower prevalence of the genus *Bacteroides* and other pro-inflammatory bacteria associated with Western dietary patterns. These changes are attributed to the high intake of fermentable substrates, which increases production of short-chain fatty acids, especially butyrate. Butyrate exerts systemic anti-inflammatory effects, including through inhibition of histone deacetylases, thereby contributing to modulation of the epigenetic pathways involved in inflammation and in glucose and lipid metabolism [14].

Recent clinical trials have begun to map microbial shifts directly onto vascular outcomes. In the crossover trial of ischemic heart disease patients by Djekic et al., the transition to a vegetarian diet significantly modified the relative abundance of bacterial genera within the families *Ruminococcaceae*, *Lachnospiraceae*, and *Akkermansiaceae* [64]. Importantly, the clinical reduction in oxidized LDL-C in response to the vegetarian diet was directly associated with a baseline gut microbiota composition dominated by specific genera of *Ruminococcaceae*. This implies that individual metabolic responses to anti-inflammatory diets are highly dependent on the baseline microbiome, suggesting a potential role for personalized, microbiota-guided precision nutrition [49].

Additionally, the OMNIVEG study confirmed that shifting to a vegan Mediterranean diet alters fecal short-chain fatty acids and plasma metabolites (such as L-carnitine and acylcarnitine metabolites), reflecting a decrease in the systemic circulating pool of metabolites that damage the vascular endothelium [65]. The host-microbe metabolic interaction provides a reasonable explanation for the rapid improvements in blood pressure, arterial stiffness, and systemic low-grade inflammation observed when transitioning to plant-based models.

Comparative analyses between omnivorous and vegan diets summarize the impact of these models on markers of metabolic syndrome. Synthesized data indicate that a typical omnivorous regimen is associated with higher fasting glucose, LDL and triglycerides, as well as increased CRP and IL-6 levels, against a background of higher consumption of saturated fats, cholesterol, refined carbohydrates, and ultra-processed foods. In contrast, vegan diets, through their high fiber and unsaturated fat content and the absence of animal fats, are associated with lower fasting glucose, improved insulin sensitivity, reduced LDL, lower triglycerides, and diminished inflammatory markers, a profile suggesting substantial anti-inflammatory and cardioprotective potential [6]. These findings may suggest that reducing the inflammatory load of the diet represents a pragmatic direction in the management strategy for metabolic syndrome, although long-term randomized interventions are needed to confirm clinical effects.

### 3.2. Pro-Inflammatory Dietary Patterns and Consumption of Ultra-Processed Foods

The structure of pro-inflammatory dietary patterns is characterized by high intake of processed and ultra-processed foods rich in saturated fats, simple sugars, and additives, together with low consumption of whole plant foods. In contrast to plant-based models, these patterns frequently include processed red meat, other processed products, sweetened beverages, and added sugar, elements explicitly classified as foods with pro-inflammatory potential in empirical dietary indices of inflammation. These components are characteristic of the so-called Western dietary pattern, which is associated with the promotion of inflammation and unfavorable effects on the immune system and intestinal microbiota [66]. While Mediterranean-type diets correlate with reduced low-grade inflammation, the Western pattern is distinguished by a systemic pro-inflammatory impact. Increased consumption of processed red meat, other processed foods, sugar, and carbonated beverages is part of a dietary profile that favors increased concentrations of high-sensitivity C-reactive protein (hsCRP) and interleukin-6 (IL-6), major biomarkers of chronic low-grade inflammation. The analysis of an empirical anti-inflammatory diet index built on 17 components showed that low scores, corresponding to patterns with high intake of such pro-inflammatory foods, are associated with relatively increased hsCRP values, whereas a higher score, reflecting a shift toward anti-inflammatory foods, correlates with progressive decreases in hsCRP. In the same study, moving from lower to higher index categories was associated with gradual reductions in IL-6, soluble TNF receptors (TNF-R1, TNF-R2), and hsCRP, suggesting that a high load of pro-inflammatory foods contributes to the maintenance of a persistent inflammatory environment. The Western pattern, dominated by ultra-processed foods, is characterized not only by an imbalanced nutritional profile, but also by negative effects on the intestinal microbiota. In contrast to Mediterranean and plant-based models, which support a diverse and anti-inflammatory microbial profile, this dietary pattern has been linked to disruption of the intestinal ecosystem and to a pro-inflammatory impact, with consequences for the immune response and mucosal barrier integrity [66,67]. In the context of metabolic syndrome, such microbiota changes may amplify lipopolysaccharide-related endotoxemia and fuel the inflammation-metabolic dysfunction cycle described earlier [6]. Another relevant element in pro-inflammatory patterns is the increased intake of saturated fats and certain forms of omega-6 fatty acids, which are frequently found in ultra-processed foods rich in animal fats and refined oils. Analysis of nutrient clusters identified a group of nutrients that includes saturated fatty acids and some n-6 polyunsaturated fatty acids associated with pro-inflammatory responses and increased LDL cholesterol. Although the same cluster also includes fatty acids with beneficial effects, the inclusion of saturated fatty acids reflects an eating behavior centered on overall high fat intake, characteristic of diets rich in ultra-processed foods, with the potential to worsen the cardiometabolic profile and inflammatory status [9]. In contrast to dietary models rich in polyphenols, fiber, and unsaturated fatty acids, pro-inflammatory patterns are notable for high energy density, low fiber content, and high intake of simple carbohydrates. Studies synthesized in a review on metabolic syndrome showed that typical omnivorous diets associated with the consumption of processed foods and simple sugars led to higher fasting glucose, LDL cholesterol, and triglyceride levels, as well as increased CRP and IL-6 values compared with vegan diets. This profile reflects the cumulative effect of pro-inflammatory foods on glucose metabolism, lipid metabolism, and systemic inflammation. At the same time, urban development and lifestyle changes toward diets rich in ultra-processed foods and poor in plant components are identified as major determinants of the increasing prevalence of metabolic syndrome in both developed countries and low- and middle-income countries. Diets high in ultra-processed products, saturated fats, and added sugars favor central obesity, insulin resistance, and dyslipidemia, key elements of a pro-inflammatory and prothrombotic environment [6,7]. In this context, directing nutritional interventions toward systematic reduction in ultra-processed food consumption and Western dietary patterns becomes an essential component of strategies for the prevention and control of cardiometabolic diseases [6,67,68]. Data from a prospective cohort study of older adults suggest that a higher dietary inflammatory index (DII) score, indicating a pro-inflammatory diet, was associated with increased risks of cardiovascular events and cardiovascular mortality, the effects being partially mediated by clinical conditions such as impaired renal function, abnormal ABI, and hyperhomocysteinemia, which may suggest mechanisms through which dietary patterns influence clinical outcomes. These observations appear to confirm the link between dietary inflammatory markers and cardiovascular consequences, although causal interpretation remains limited by the observational nature of the data [69].

### 3.3. Dietary Inflammatory Indices and Diet Quality Scores

The conceptualization of dietary patterns through quantitative indices allows estimation of the inflammatory or anti-inflammatory potential of the diet based on the consumption of food groups or nutrients, in close relationship with systemic biomarkers of low-grade inflammation. A representative example is the 17- component empirical anti-inflammatory diet index (eADI-17), developed in a subsample of older Swedish men, in which food-frequency questionnaires and four plasma biomarkers of chronic inflammation were used: hsCRP, IL-6, TNF-R1, and TNF-R2. Construction of eADI-17 first involved deriving a “composite inflammatory factor” through principal component analysis of the four biomarkers, followed by application of Lasso regression to identify food groups associated with a pro- or anti-inflammatory status. Based on these results, a score was developed to classify each person’s intake into consumption tertiles for foods with anti-inflammatory or pro-inflammatory potential; the final score represents the net balance between these two categories, with higher values indicating a relatively higher intake of anti-inflammatory foods [66,67]. Validation in an independent replication set confirmed a significant association of the score with hsCRP, IL-6, TNF-R1, and TNF-R2 levels, suggesting that eADI-17 can predict low-grade systemic inflammation. The magnitude of the Spearman correlations between eADI-17 and biomarkers ranged from −0.17 for hsCRP to −0.28 for TNF-R1, indicating a moderate inverse relationship between the anti-inflammatory score and plasma inflammation. By comparison, a previous anti-inflammatory index (AIDI-20) showed a similar correlation with hsCRP, whereas a pro-inflammatory index (EDII) showed positive correlations with hsCRP, IL-6 and TNF-R2, but of slightly lower magnitude [43]. These data show that the simultaneous inclusion of several biomarkers may increase the sensitivity of dietary inflammation indices and support the use of composite scores for assessing the inflammatory load of the diet. Analysis of eADI-17 categories revealed a clear gradient: participants in the quintiles with higher scores had relatively lower concentrations of all four biomarkers, with reductions of up to 29% for hsCRP and 19% for TNF-R1 in the discovery set, and up to 28% and 14%, respectively, in the replication set. The authors’ interpretation emphasizes that, although hsCRP is frequently used as a marker of inflammation, the stronger correlations of eADI-17 with IL-6, TNF-R1 and TNF-R2 suggest that these biomarkers may be more sensitive indicators of diet-induced inflammatory response, concluding that the use of a multiple-marker panel is preferable in diet inflammation studies. To extend applicability to other cohorts, eADI-17 was adapted into a 15-component instrument (eADI-15), because questions on seeds and legumes were absent from the food-frequency questionnaire used in large population studies. The results for eADI-15 were practically similar to those for eADI-17, and, in the replication group, each 4.5-point increase in the eADI-15 score was associated with decreases of 13% in hsCRP, 7% in IL-6, 9% in TNF-R1, and 10% in TNF-R2. These findings suggest that more compact score structures can preserve predictive ability for low-grade inflammation, facilitating the practical use of dietary indices in nutritional epidemiology [67]. Another methodological approach focused on identifying nutrient clusters associated with the dietary inflammatory index (E-DII) in patients with type 2 diabetes mellitus and prediabetes. Factor analysis identified five nutritional clusters: antioxidant-mineral, protein-vitamin B complex, fatty acids, plant lipids and immunomodulatory micronutrients, reflecting co-ingestion patterns that contribute to E-DII variability. Ordinal logistic regression models showed that higher intake from the antioxidant–mineral cluster, which includes fiber, folic acid, vitamin C, magnesium, iron and carbohydrates, was associated with a significantly lower probability of being in the highest E-DII tertile, with an odds ratio of 0.08. The fatty-acid cluster and the immunomodulatory micronutrient cluster were also associated with reduced odds of a high E-DII, suggesting that nutrient profiles rich in fiber, vitamins and minerals with antioxidant and immunomodulatory roles correlate with a lower inflammatory potential of the diet. Analysis of the antioxidant-mineral cluster highlights the role of magnesium and iron as cofactors for antioxidant enzymes, as well as the association of magnesium intake with lower hsCRP, IL-6 and TNF-α levels, whereas folic acid, vitamin C and iron contribute to immune function, regulation of oxidative stress and maintenance of arterial and renal health. These results reinforce the idea that diet-quality scores based on inflammatory load can be refined by integrating information on nutrient groups with convergent effects on inflammation and oxidative stress [68]. Overall, the development and validation of indices such as eADI-17, eADI-15, and E-DII illustrate a quantitative approach to the diet-inflammation interface, which can be used to evaluate and guide dietary interventions in metabolic syndrome and cardiometabolic diseases by directing intake toward foods and nutrients with a demonstrated anti-inflammatory profile [67,68].

### 3.4. Macronutrient Quality and Dietary Glycemic Load

Macronutrient quality and the structure of dietary carbohydrates directly determine glycemic load and, implicitly, the impact on low-grade inflammation and the metabolic parameters involved in metabolic syndrome. Well-structured plant-based dietary patterns are characterized by a high intake of complex carbohydrates and fiber derived from whole grains, legumes, and vegetables, which stabilizes blood glucose and improves insulin sensitivity, suggesting adjuvant potential in the metabolic management of patients with type 2 diabetes [6,70]. Systematic reviews have shown that specific dietary supplements improved metabolic syndrome components, with key options including phytosterols, probiotics/synbiotics, nanocurcumin, and phytosterols. Nanocurcumin supplementation proved to alleviate metabolic syndrome components and cardiometabolic-related parameters, including glycemic control, lipid profile, anthropometric measures, blood pressure, oxidative stress markers, and inflammatory biomarkers [71,72,73].

Micronutrients and bioactive compounds from plant-derived foods exert a wide range of effects on inflammatory pathways, oxidative stress and the immune response, with direct relevance for metabolic and cardiometabolic diseases. An analysis of nutrient clusters centered on the E-DII dietary inflammatory index identified an antioxidant-mineral group composed of vitamin C, folate, magnesium and iron, in which higher intake was associated with a significantly reduced probability of having a high E-DII score, reflecting a dietary profile with a lower inflammatory load. These micronutrients are derived predominantly from citrus fruits, leafy vegetables, legumes, and whole grains, emphasizing the importance of whole foods rich in vitamins and minerals for the modulation of chronic inflammation [69].

Other classes of nutraceuticals include carotenoids and isoprenoids (lycopene, lutein, astaxanthin), with roles in antioxidant defense, immune modulation, and control of lipid peroxidation, as well as bioactive peptides and amino acid derivatives from marine sources or fermented foods involved in immunometabolic regulation, epithelial repair, and control of oxidative stress. In addition, long-chain omega-3 fatty acids such as EPA and DHA are precursors of pro-resolving lipid mediators (resolvins, protectins, maresins), which actively participate in the resolution of inflammation by reducing neutrophil infiltration, promoting non-inflammatory efferocytosis and supporting macrophage phenotypic transition [74]. These examples illustrate how micronutrients and bioactive compounds can be integrated into anti-inflammatory nutritional interventions, but also the need for well-designed randomized trials with standardized interventions and validated inflammatory biomarkers, as emphasized by recent analyses of nutraceuticals [75,76]. Table 4 synthesizes the biochemical characteristics of meta-inflammation and thrombosis under the impact of omnivore vs. vegan/Mediterranean diets.

## 4. The Gut–Microbiota–Inflammation–Heart Axis

### 4.1. The Gut Microbiota as a Metabolic and Immunological Organ

The gut microbiota is described as a metabolic organ capable of transforming dietary nutrients into bioactive metabolites with systemic effects, including on the heart and blood vessels, which justifies its integration into the concept of the gut–heart axis. Imbalances in microbial composition, defined as dysbiosis, are correlated with the progression of atherosclerosis, hypertension, arrhythmia, and heart failure, whereas a balanced microbial ecosystem is associated with more favorable cardiometabolic profiles [81,82]. Metabolites derived from microbial activity illustrate the metabolic role of the gut microbiota [40]. TMAO, produced from choline, carnitine, and lecithin, has been associated in numerous observational studies with adverse cardiovascular outcomes, including major events and progression of heart failure, although causality remains controversial. TMAO is a gut-microbiome-derived metabolite whose complex role involves dietary precursor generation, hepatic oxidation, and multi-system pathology. Human and experimental studies linked elevated TMAO to cardiovascular disease, renal dysfunction, autism disorders, neurodegenerative and metabolic disorders, although physiological baseline functions also include protein stabilization. TMAO has a complex role in experimental studies, acting both as a detrimental pro-inflammatory mediator in chronic diseases and as an adaptive organic osmolyte/protein stabilizer. Its ultimate impact depends heavily on baseline tissue health, disease context, and concentration thresholds [83,84].

Short-chain fatty acids (SCFAs), generated through fermentation of indigestible dietary fiber, have anti-inflammatory and cardio-energetic effects, while secondary bile acids, indoles, PAGln and microbially derived branched-chain amino acids complete the spectrum of mediators of the gut–heart axis [85]. SCFAs such as acetate, propionate and butyrate are produced in the colon through fermentation of resistant starch, pectin, inulin and other indigestible complex carbohydrates. After absorption, propionate and acetate are metabolized in the liver and enter systemic circulation, acting on G-protein coupled receptors GPR41/FFAR3 and GPR43/FFAR2 on endothelial cells, immune cells and adipose tissue, as well as via the inhibition of histone deacetylases. Through these mechanisms, SCFAs strengthen intestinal barrier integrity, regulate lipid and glucose homeostasis, and reduce systemic inflammation [85,86]. Experimental data show that SCFAs improve endothelial dysfunction, lower blood pressure, and attenuate cardiac hypertrophy and fibrosis, while butyrate increases HDL levels and reduces LDL, suggestive of cardiovascular protection through the improvement of lipid metabolism and reduction in oxidative stress [40,87]. Indoles, such as indole-3-propionic acid (IPA), resulting from the metabolism of tryptophan by specific bacteria, exert antioxidant and anti-inflammatory effects through activation of the AhR receptor and modulation of NAD metabolism. IPA has been associated with reduced oxidative stress and improved endothelial integrity, protecting cardiomyocytes against lipid peroxidation. In conditions of dysbiosis, however, tryptophan metabolism shifts toward kynurenine and indoxyl sulfate, metabolites that induce oxidative stress and endothelial apoptosis through activation of the AhR–p38–MAPK axis, compromising vascular integrity and emphasizing the context-dependent role of the microbiota [86,87]. Secondary bile acids represent another example of microbiota metabolic function. In the intestine, bacteria transform primary bile acids into secondary acids, modifying the size and composition of the circulating pool and, implicitly, its signaling potential. Bile acids interact with nuclear receptors (FXR, PXR, VDR) and membrane receptors (TGR5, muscarinic receptors), which are also expressed in endothelial cells, smooth muscle cells, and cardiomyocytes, where they influence cholesterol and glucose metabolism, vascular tone, and inflammatory responses. At physiological levels, activation of FXR and TGR5 is vasculoprotective, promoting NO-dependent vasodilation, improved endothelial function, and anti-inflammatory and hypolipidemic effects. Excess of certain secondary bile acids, such as deoxycholic acid, may, however, induce oxidative injury, mitochondrial dysfunction, endothelial damage, and reduced cardiac contractility, highlighting the duality of these metabolites [1,88]. On the immunological component, microbial products modulate inflammatory pathways central to cardiometabolic pathology. SCFAs and indole derivatives have anti-inflammatory and antioxidant properties; butyrate and propionate inhibit HDAC, restore the Treg/Th balance, and suppress NF-κB-dependent cytokine expression, while increasing antioxidant enzymes such as catalase and glutathione peroxidase. IPA acts as a free-radical scavenger and reduces lipid peroxidation, contributing to cardiomyocyte protection. In contrast, TMAO activates NF-κB, increases the production of IL-1β and IL-6, and stimulates expression of endothelial adhesion molecules VCAM-1 and ICAM-1, favoring immune-cell recruitment and endothelial injury. From the perspective of oxidative stress, TMAO increases ROS formation through activation of NADPH oxidase and decreased expression of mitochondrial antioxidant enzymes such as SOD2, through inhibition of the sirtuins SIRT1 and SIRT3. This pro-oxidant balance is associated with reduced NO bioavailability and endothelial dysfunction. By contrast, SCFAs and indoles, including IPA, display robust antioxidant activity through stimulation of catalase and glutathione peroxidase as well as through the neutralization of free radicals [89]. Signaling mediated by FXR and TGR5 by certain bile-acid species may also modulate redox balance, with FXR activation being linked to attenuation of ET-1-mediated vasoconstriction through AMPK and improvement of endothelial function [90]. Integrative data on these metabolites support the conceptualization of the microbiota as a metabolic and immunological organ, positioned at the interface between diet, inflammation, oxidative stress and cardiovascular function, and provide the foundation for the nutritional and microbiota-directed interventions discussed below [81].

### 4.2. Diet-Derived Microbial Metabolites with Cardiometabolic Impact

Diet-derived microbial metabolites represent a central node of the gut–heart axis because they connect the structure of food intake with inflammation, oxidative stress, thrombosis, and cardiac remodeling. The main metabolites include several major classes: TMA/TMAO, PAGln, secondary bile acids, amino acids and SCFAs, as well as indoles, each with specific dietary sources, distinct microbial pathways and molecular targets relevant to cardiometabolic pathology. TMAO derives from TMA, a product of the metabolism of choline, phosphatidylcholine and carnitine, nutrients rich in trimethylamine moieties present in red meat, eggs and fish. After absorption, TMA is oxidized in the liver by FMO3 to TMAO. Elevated circulating TMAO levels have been correlated with interference with reverse cholesterol transport, deposition of cholesterol in the arterial wall, and acceleration of atherosclerosis. At the vascular level, TMAO is associated with endothelial dysfunction, oxidative stress, inflammatory activation, and platelet hyperreactivity, promoting vascular injury and atherothrombosis [1,6,89]. Animal models supplemented with TMAO, choline, or carnitine show increased formation of macrophage foam cells and accelerated development of atherosclerotic plaques. In diabetic mice or mice with metabolic alterations, TMAO is elevated; its reduction through FMO3 knockdown improves insulin tolerance and reduces cardiovascular complications. The pro-inflammatory and pro-oxidant action of TMAO has been thoroughly described. Elevated plasma levels activate NF-κB signaling, increase IL-1β and IL-6 production, and upregulate endothelial adhesion molecules such as VCAM-1 and ICAM-1, favoring cellular infiltration and endothelial injury. TMAO primes the NLRP3 inflammasome, amplifying inflammatory responses, and stimulates the formation of reactive oxygen species through activation of NADPH oxidase and reduction in mitochondrial antioxidant systems, including SIRT1/3 and SOD2. These changes reduce nitric oxide bioavailability and worsen endothelial dysfunction. PAGln is produced through the catabolism of phenylalanine by the microbiota followed by host conjugation; its targets include alpha2A, alpha2B, and beta2 adrenergic receptors. This metabolite increases platelet reactivity and thrombotic risk, representing a direct mediator between protein intake and thrombosis through modulation of adrenergic pathways. Amino acids, especially BCAAs derived from protein-rich diets, are involved in the activation of the mTOR pathway and mitochondrial routes, with effects on hypertrophy, fibrosis, and energy disturbances, particularly in heart failure with preserved ejection fraction. Secondary bile acids result from deconjugation and dihydroxylation of primary bile acids by intestinal bacteria [27]. They modulate lipid metabolism, vascular tone, contractility and inflammation through interactions with FXR, TGR5, PXR, VDR and muscarinic receptors. Activation of FXR and TGR5 is linked to anti-inflammatory and hypolipidemic effects, with improved endothelial function; however, excessive accumulation of secondary bile acids can induce oxidative lesions and endothelial dysfunction, emphasizing the dose- and species-dependent nature of these mediators. SCFAs such as acetate, propionate, and butyrate are produced through anaerobic fermentation of dietary fiber and resistant starch [40]. They act on GPR41/43 receptors and inhibit HDAC, with complex effects on mitochondrial energetics, inflammation, and cardiac remodeling. SCFAs improve endothelial dysfunction, lower blood pressure, and attenuate cardiac hypertrophy and fibrosis; butyrate in particular increases HDL and reduces LDL, supporting cardiovascular protection through improved lipid metabolism and reduced oxidative stress. Through HDAC inhibition and rebalancing of the Treg/Th ratio, butyrate and propionate suppress NF-κB-dependent cytokine expression and increase the activity of antioxidant enzymes, including catalase and glutathione peroxidase. Indoles, such as indole-3-propionic acid (IPA), are generated from dietary tryptophan. IPA activates the AhR receptor and modulates NAD metabolism, exerting antioxidant and anti-inflammatory effects, and has been described as protective against diastolic dysfunction [86]. IPA acts as a free radical scavenger, protecting cardiomyocytes against lipid peroxidation and improving endothelial integrity. In conditions of dysbiosis, however, tryptophan metabolism shifts toward kynurenine and indoxyl sulfate, metabolites that induce oxidative stress and endothelial apoptosis through activation of the AhR–p38–MAPK axis, compromising vascular integrity. The dietary context and nutritional interventions shape these metabolic fluxes. Mediterranean and plant-based regimens, rich in fiber, polyphenols, and omega-3 fatty acids, increase SCFA production and favorably modulate bile acid signaling, improving lipid metabolism, endothelial function, and anti-inflammatory status. Increased fiber intake augments colonic fermentation, increasing systemic acetate and butyrate, supporting mitochondrial biogenesis, oxidative phosphorylation, and inhibition of pro-inflammatory cytokine production. Interventions with probiotics and prebiotics can promote SCFA-producing taxa and remodel the bile-acid pool; however, results regarding TMAO remain mixed, with some trials failing to reduce plasma TMAO through probiotics or inulin. By contrast, restriction of red meat and choline-rich foods is proposed as a strategy for reducing TMA and TMAO, alongside pharmacological approaches targeting the microbial enzymes CutC/D [89]. Recent epidemiological studies show that empirical indices of the inflammatory and insulinemic potential of the diet are associated with a less favorable cardiometabolic profile, including higher insulin and C-peptide levels and lower HDL, which may suggest an additional pathway through which diet influences cardiometabolic risk. These observations appear to confirm that dietary modifications reducing inflammatory and insulinemic potential may have beneficial effects on biomarkers relevant to cardiovascular and metabolic diseases [91].

### 4.3. Nutritional Interventions Directed Toward Microbiota Modulation

Nutritional interventions directed toward microbiota modulation focus on adjusting the composition and function of the intestinal ecosystem through changes in dietary intake and the use of probiotics, prebiotics, synbiotics, or more direct therapies such as fecal microbiota transplantation. Mediterranean and plant-based regimens, characterized by high intake of fiber, polyphenols, and omega-3 fatty acids, are associated with increased production of SCFAs and favorable modulation of bile-acid signaling, which contribute to improved lipid metabolism, endothelial function, and anti-inflammatory status. High fiber intake amplifies colonic fermentation, increasing systemic acetate and butyrate levels, which support mitochondrial biogenesis, oxidative phosphorylation, and suppression of pro-inflammatory cytokine production. Probiotics and prebiotics represent pragmatic strategies for modulating microbiota to reduce cardiometabolic risk. Nutritional interventions that include probiotics and prebiotics have demonstrated modest improvements in some risk markers, such as blood pressure, lipid profile, and glycemic parameters, and inflammatory indices, although results vary considerably according to formulation, dose, and study population. Mechanistically, these approaches promote SCFA-producing taxa and remodel the bile acid pool, with potential influence on FXR/TGR5 signaling, which is relevant for lipid homeostasis and inflammatory response [89]. Prebiotics, by stimulating the growth of fermentative species, increase butyrate and acetate production, which may support intestinal barrier integrity and reduce systemic inflammation. In metabolic syndromes and metabolism-related liver diseases, synbiotics (combinations of probiotics and prebiotics) may provide additional benefits by simultaneously supplying favorable microorganisms and fermentable substrates. Data synthesized in patients with metabolic dysfunction-associated steatotic liver disease (MASLD) indicate that probiotics may restore microbial balance, reduce intestinal permeability, and decrease the translocation of endotoxins such as lipopolysaccharides, thereby diminishing systemic inflammation and liver injury. Certain probiotic strains influence bile acid metabolism, which is important for lipid homeostasis, and may improve insulin sensitivity and hepatic steatosis by regulating cytokines and immune-cell populations in the liver. Prebiotics support microbial diversity and the production of SCFAs, especially butyrate, which reduces hepatic fat accumulation and improves insulin sensitivity through effects on gene expression in the liver and muscle, while also strengthening the intestinal barrier by stimulating mucins and tight-junction proteins [92]. Analyses of microbiota-directed interventions in obesity, type 2 diabetes mellitus, metabolic syndrome, and non-alcoholic fatty liver disease show a broad range of outcomes. Probiotics have been associated with lower CRP, improved glycemic control, lipid profile, and body composition in people with obesity or type 2 diabetes. Synbiotics and prebiotics have shown potential to improve intestinal barrier integrity and attenuate hepatic inflammation in NAFLD. Fecal microbiota transplantation from lean donors improved insulin sensitivity and increased the abundance of butyrate producers in some trials involving individuals with metabolic syndrome or insulin-resistant obesity, although other studies did not observe significant glycemic benefits. Dietary interventions rich in fiber and legumes led to decreases in HbA1c and LDL, mediated by changes in fiber-degrading taxa and microbial metabolites, including SCFAs and bile acids [93]. Targeted approaches directed at the TMA/TMAO pathway aim to reduce a metabolite considered pro-atherothrombotic.

Restricting foods rich in choline and carnitine, such as red meat and certain organ meats, has been proposed to reduce microbial TMA production. In parallel, pharmacological inhibition of microbial CutC/D enzymes has been identified as a novel therapeutic strategy. However, interventions with probiotics and prebiotics such as inulin have not consistently reduced circulating TMAO levels in controlled trials, highlighting the complexity of this metabolic pathway and the current limitations of single nutritional interventions targeting TMAO [89]. A narrative synthesis of human studies suggests that microbiota-directed therapies produce improvements that are generally modest and domain-specific rather than a global reversal of metabolic dysregulation. Response variability is attributed to baseline microbiota composition, dietary habits, and host characteristics, while umbrella reviews indicate that, although microbiota interventions frequently influence metabolic indicators, the robustness and consistency of evidence are still developing. In this context, the combination of dietary patterns rich in fiber and polyphenols with the selective use of probiotics, prebiotics, or synbiotics, adapted to the individual metabolic risk profile, appears to be a promising direction for modulating the gut–microbiota–inflammation–heart axis [89,92,93]. Table 5 classifies the main microbial metabolites involved in cardiovascular pathology and their biochemical targets.

## 5. Integrated Anti-Inflammatory Strategies in the Prevention and Progression of Cardiovascular Diseases and Metabolic Syndrome

### 5.1. Diet, Inflammation, and the Risk of Cardiovascular Events

The relationship between diet quality, low-grade inflammation, and the incidence of cardiovascular events is mediated by a set of metabolic, endothelial, and thrombotic mechanisms. Dietary patterns rich in saturated fats, cholesterol, simple carbohydrates, and ultra-processed foods favor the onset and progression of metabolic syndrome, which amplifies systemic inflammation and thrombotic risk, with direct consequences for myocardial infarction, stroke, and venous thromboembolism [6]. In contrast, plant-based dietary patterns, including vegan and Mediterranean diets, are associated with a more favorable metabolic and inflammatory profile, which may reduce the likelihood of these events [6,55]. Metabolic syndrome, strongly influenced by diet, represents the link between nutrition and cardiac and vascular pathology. Hyperglycemia, central obesity, dyslipidemia, and hypertension, which define this syndrome, often arise against a background of urbanization, sedentary behavior, and increased consumption of ultra-processed foods. Adipocytes and macrophages in adipose tissue release pro-inflammatory cytokines, generating chronic low-grade inflammation that accelerates atherogenesis and deteriorates endothelial function. In this context, diet acts not only as a metabolic risk factor but also as a determinant of the inflammatory status that influences atherosclerotic plaque stability and predisposition to thrombosis [33,50]. On the thrombotic component, a diet that favors the development of metabolic syndrome indirectly contributes to an increased risk of cardiovascular events by inducing a prothrombotic state. Visceral obesity and insulin resistance, sustained by patterns rich in saturated fats and refined sugars, induce increased secretion of cytokines and adipokines, which stimulates activation of coagulation factors and increases PAI-1. This imbalance between coagulation and fibrinolysis favors both arterial thrombosis, with myocardial infarction and stroke, and venous thromboembolism, including deep vein thrombosis and pulmonary embolism. Thus, diet-induced inflammatory and prothrombotic burden translates into a cumulative increase in the risk of major cardiovascular events [6]. Anti-inflammatory dietary patterns are distinguished by their effects on inflammatory markers and endothelial function, key mechanisms in the prevention of cardiovascular events. The Mediterranean diet, especially the “green” variant enriched with polyphenols from Mankai, walnuts, and green tea, has demonstrated reductions in intrahepatic fat, diastolic blood pressure, LDLcholesterol, and C-reactive protein in individuals with abdominal obesity or dyslipidemia. Its bioactive components, such as monounsaturated fatty acids, omega-3 fatty acids, fiber, nitrates, and polyphenols, attenuate oxidative stress by neutralizing free radicals, inhibit the NF-kB cascade, and increase nitric oxide bioavailability by stimulating eNOS expression, thereby improving flow-mediated dilation and reducing arterial stiffness. These modifications in endothelial function represent a proximal mechanism through which the Mediterranean diet may lower the probability of ischemic events [1,33,50]. Plant-based diets, including vegetarian and vegan patterns, exert similar effects on the vasculature. A plant-based dietary counseling program in patients with peripheral arterial disease and hypercholesterolemia produced, after four months, significant reductions in total cholesterol and LDL, together with improved flow-mediated dilation and reduced pulse-wave velocity, indicating lower arterial stiffness [55]. These effects overlap with general metabolic benefits: improved insulin sensitivity, lower fasting glucose, reduced LDL and triglycerides, and decreased inflammatory markers such as CRP and IL-6 in individuals following well-structured vegan diets [6]. By reducing inflammation and dyslipidemia simultaneously, these dietary patterns may decrease atherosclerotic burden and plaque instability, with a direct impact on the risk of myocardial infarction and stroke [6,55]. The detailed nutritional composition of vegan diets provides an additional explanation for the observed cardiovascular effects. Replacing saturated fats with polyunsaturated fatty acids, especially PUFAs, lowers LDL cholesterol without adversely affecting HDL, while the inclusion of phytosterols, which compete with cholesterol for intestinal absorption, further reduces LDL. Plant-based omega-3 fatty acids support cardiovascular health and reduce inflammation, while replacing heme iron with non-heme sources may diminish oxidative stress resulting from free-radical formation and lipid peroxidation. Together, these lipid and redox modifications contribute to reducing the risk of atherosclerosis and, implicitly, coronary and cerebrovascular events [6]. Low-grade inflammation induced by diet can also be quantified using dietary inflammatory scores, which have been shown to be associated with cardiovascular and non-cardiovascular mortality. An observational study showed that higher Dietary Inflammatory Index scores correlate with a significant increase in all-cause and cardiovascular mortality, including sex-specific associations, with higher relative risks among women in the upper score categories. These data support the idea that not only isolated nutrients, but the overall inflammatory pattern of the diet, influences the risk of fatal events [100,101]. Another level of integration is provided by empirical anti-inflammatory indices, such as eADI-17, which quantify the ratio between pro- and anti-inflammatory foods. An increase in the eADI-17 score was associated with decreases of up to 29% in hsCRP and 19% in TNF-R1, while the adapted eADI-15 version showed similar reductions in hsCRP, IL-6, and soluble TNF receptors [8]. Although these studies were conducted mainly in older populations, the results suggest that diets with high anti-inflammatory scores may reduce the systemic inflammatory burden associated with cardiovascular events, offering a practical tool for evaluating and guiding nutritional interventions [67,101]. Overall, the evidence indicates that dietary patterns low in saturated fats and ultra-processed foods and rich in fiber, polyphenols, and unsaturated fatty acids reduce low-grade inflammation and improve endothelial function, lipid profile, and thrombotic status, translating into a lower risk of major cardiovascular events [6,55,67,100]. A recent pilot clinical study showed that supplementation with *Limosilactobacillus reuteri* ATCC PTA 6475 was associated with a reduction in body fat percentage and favorable changes in gut microbiota composition, suggesting that microbiota-targeted interventions may partially contribute to reducing the inflammatory and metabolic burden involved in cardiovascular risk [102]. Although these conclusions are preliminary and require confirmation in controlled studies with longer durations and larger samples, they indicate a complementary pathway through which dietary adjustments and microbiota modulation may influence prothrombotic phenotypes and systemic inflammation associated with cardiovascular events.

### 5.2. Endothelial Dysfunction and the Effects of Dietary Patterns

Endothelial dysfunction, a central link between metabolic risk factors and cardiovascular events, is strongly influenced by dietary profile. A healthy endothelium maintains vascular homeostasis through the production of nitric oxide (NO) from L-arginine via eNOS and through the nitrate–nitrite–NO pathway, regulates vasomotor tone, inhibits platelet aggregation, and reduces the expression of adhesion molecules such as VCAM-1 and ICAM-1, thereby preventing leukocyte recruitment and excessive thrombogenesis [103]. This balance is lost in oxidative stress and chronic inflammation: ADMA, an endogenous competitive inhibitor of NOS, accumulates due to increased PRMT activity and reduced degradation through DDAH, whose functions are impaired by oxidative stress, thereby decreasing NO synthesis and sustaining the vicious cycle of endothelial dysfunction [55]. Dietary patterns influence this pathology both by modifying global metabolic risk and direct effects on the endothelium [6]. The DASH diet, rich in fruits, vegetables, whole grains, and low-fat dairy products and low in sodium and saturated fats, is one of the best documented examples. Recent meta-analyses show that DASH interventions increase flow-mediated dilation (FMD) by approximately 2.5% at 6 months and 3.1% at 18 months compared with standard care, improvements that translate into estimated reductions in cardiovascular events of 20–32% and 25–40%, respectively. These effects are attributed to a diet dense in minerals (potassium, magnesium, calcium), fiber, and antioxidants, and low in sodium, with possible contributions from increased intake of unsaturated fats and nitrate-rich vegetables, which support NO synthesis and reduce oxidative stress. The Mediterranean diet, enriched in plant foods, fish, and olive oil, represents another model with major endothelial impact. A “green” variant, fortified with polyphenols from Mankai, walnuts, and green tea, reduced intrahepatic fat, diastolic blood pressure, LDL cholesterol, and C-reactive protein in individuals with abdominal obesity or dyslipidemia, suggesting improvement in low-grade inflammation and vascular function [55]. Key bioactive components of this model include monounsaturated fatty acids (especially oleic acid from extra-virgin olive oil), omega-3 polyunsaturated fatty acids, fiber, dietary nitrates, and a wide range of polyphenols. These compounds attenuate oxidative stress by scavenging free radicals, inhibit NF-kB-dependent inflammatory cascades, and increase NO bioavailability by stimulating eNOS expression and preventing eNOS uncoupling, mechanisms that support endothelium-dependent dilation and reduce arterial stiffness [6,55]. Plant-based patterns provide direct interventional evidence regarding endothelial function. In a randomized study of patients with peripheral arterial disease and hypercholesterolemia, a plant-based dietary counseling program, compared with the absence of specific dietary recommendations, produced after four months a 7.6% reduction in total cholesterol and a 13.6% reduction in LDL, accompanied by significant improvement in FMD and reduced pulse-wave velocity [55]. These changes suggest increased NO production and reduced arterial stiffness, reflecting improvement in endothelial dysfunction. In parallel, vegan and vegetarian diets reduce LDL, triglycerides, and inflammatory markers such as CRP through their low content of saturated fat and cholesterol and their high intake of fiber, plant-based omega-3 fatty acids, and polyphenols, contributing to long-term endothelial protection [6].

Studies on other dietary patterns further support the role of diet in modulating endothelial function. The Nordic diet, characterized by increased intake of fatty fish, berries, root vegetables, whole grains (oats, rye), and rapeseed oil, improved vascular markers, including reduced hsCRP and improved blood pressure, in trials involving individuals with metabolic syndrome. A smaller study demonstrated that a short-term Nordic intervention increases endothelium-dependent vasodilation at the microvascular level in both young and older adults, probably through the antioxidant effects of flavonoids and anthocyanins from berries, increased NO production induced by omega-3 fatty acids, and stimulation of short-chain fatty acid production through the intake of beta-glucan-rich whole grains. The traditional Japanese diet (Washoku) and the Paleolithic diet also show endothelial potential, although direct evidence based on FMD is limited. Washoku is characterized by regular consumption of omega-3-rich fish, soy foods, and green tea, with meta-analyses of green tea intervention studies showing improvement in brachial FMD, probably through activation of eNOS via the PI3K/Akt pathways by catechins, especially epigallocatechin gallate, and antioxidant protection against endothelial oxidative stress. For the Paleolithic diet, a meta-analysis of randomized trials indicates a decrease in CRP, suggesting a possible anti-inflammatory effect with indirect benefits on the endothelium, probably through the high intake of antioxidant-rich fruits and vegetables and omega-3 from fish [24].

In contrast, dietary patterns rich in ultra-processed foods and saturated fats promote metabolic syndrome and an inflammatory and procoagulant environment that accelerates endothelial deterioration. Central obesity and insulin resistance, sustained by such diets, increase ROS production, activate the MAPK and NF-κB pathways, and reduce insulin-stimulated NO synthesis, which upstream leads to increased ICAM-1 and VCAM-1, vasoconstriction, and hypertension [6]. In this context, shifting from a Western dietary pattern to plant-based, Mediterranean, or Nordic dietary patterns represents a central nutritional strategy for preventing and reversing endothelial dysfunction and, implicitly, reducing the burden of cardiovascular diseases [6,24,31]. Recent observational and interventional data in cancer survivors have shown that Mediterranean-diet-based interventions may modulate systemic inflammatory profiles, reducing pro-inflammatory markers such as IL-12 and leptin, changes that may suggest a beneficial effect on mechanisms of chronic low-grade inflammation of the endothelium. These findings support the idea that optimizing dietary habits can produce measurable effects on inflammatory biomarkers relevant to vascular health [74]. Healthy dietary models display a remarkable capacity to reverse endothelial dysfunction, as documented in Table 6.

### 5.3. Integrated Dietary Approaches, Lifestyle Strategies, Role of Meal Timing and Intermittent Fasting on Weight Control in the Management of Metabolic Syndrome

Integrating dietary strategies into the management of metabolic syndrome involves combining modification of the overall dietary pattern with targeted interventions on the microbiota and diet-derived metabolites. Plant-based patterns, including vegan and Mediterranean diets, represent a central pillar because they are rich in fiber, polyphenols, and unsaturated fatty acids, while limiting saturated fats, cholesterol, and simple carbohydrates. This nutritional profile translates into integrated improvements in body weight, glycemia, lipid profile, and inflammatory markers, which are key elements in the control of metabolic syndrome [1,6,108]. Within well-structured vegan diets, increased intake of fiber and complex carbohydrates from whole grains, legumes, and vegetables stabilizes blood glucose and increases insulin sensitivity. Comparative tables of metabolic syndrome markers show that, compared with an omnivorous diet, a vegan diet is associated with lower fasting glucose, LDL cholesterol, and triglycerides, together with reductions in CRP and IL-6, owing to anti-inflammatory phytonutrients and plant-based omega-3 fatty acids. Synthesized studies indicate decreases in body mass index and waist circumference, reflecting reduced visceral adiposity, which contributes to improving insulin resistance and the thrombotic profile associated with metabolic syndrome. A meta-analysis of plant-based diets in people with obesity showed improved insulin resistance and reduced total cholesterol, HDL, and LDL, suggesting that this pattern may be used as an integrated strategy for glycemic and lipid control, even if the effect on triglycerides was not significant [1,6]. An important added value of integrated dietary approaches is their impact on the gut microbiota and microbial metabolites. Vegan diets, rich in fiber, polyphenols, and resistant starch, generate a distinct microbial profile, with increased abundance of *Prevotella* and decreased *Bacteroides* and other pro-inflammatory bacteria associated with Western diets. This remodeling favors the production of short-chain fatty acids, especially butyrate, with systemic anti-inflammatory effects through histone deacetylase inhibition and modulation of epigenetic signaling involved in inflammation and glucose and lipid metabolism [6]. In parallel, high-fiber intake and reduction in animal fats decrease the translocation of endotoxin lipopolysaccharides (LPS) and circulating levels of TMAO, a pro-atherothrombotic metabolite derived from animal proteins, thereby contributing to the reduction in low-grade inflammation and cardiometabolic risk [6,89]. Integrated dietary approaches can be expanded through the targeted use of probiotics, prebiotics, and synbiotics as adjuvants to healthy dietary patterns. Microbiota-directed nutritional interventions have demonstrated decreases in CRP and improvements in glycemic control, lipid profile, and body composition in individuals with obesity or type 2 diabetes mellitus, although the magnitude of effects varies according to formulation, dose, and population characteristics [89,93]. Prebiotics increase SCFA production and strengthen the intestinal barrier, reducing metabolic endotoxemia, whereas probiotics can remodel the bile-acid pool and FXR/TGR5 signaling, with effects on lipid homeostasis and inflammatory responses [21,25]. Synbiotics and diets rich in fermented foods, such as tempeh or kimchi, can be integrated into a vegan dietary pattern to optimize modulation of microbial metabolites and metabolic syndrome parameters [6,93]. A complementary element of these approaches is the systematic reduction in foods rich in choline and carnitine, especially red meat and organ meats, to limit microbial production of TMA and TMAO, considered pro-atherothrombotic [89]. The strategy can be implemented simultaneously with increased intake of fiber and polyphenols, typical of Mediterranean and plant-based diets, to encourage SCFA-producing taxa and a bile-acid profile with vasculoprotective signaling [6,89]. In this sense, integrating red-meat restriction with a diet rich in olive oil, nuts, legumes, whole grains, and vegetables offers a coherent nutritional framework for simultaneously reducing inflammation, dyslipidemia, insulin resistance, and thrombotic risk [6,55,89]. In clinical practice, dietary inflammatory scores such as eADI-17 can be used to guide integrated nutritional interventions. A high score, reflecting elevated intake of vegetables, whole-grain muesli, vegetable oils, nuts and seeds, and reduced intake of processed foods, has been associated with consistent decreases in hsCRP, IL-6, and soluble TNF receptors, indicating that fine adjustment of the dietary pattern can reduce the systemic inflammatory burden relevant to metabolic syndrome [82]. Combined with microbiota-directed interventions, these integrated dietary approaches offer a complex framework for managing metabolic syndrome and reducing associated cardiometabolic risk [6,82,89,93]. In addition, time-restricted eating has emerged as a promising and highly topical nutritional strategy for managing metabolic syndrome. Meal-timing synchronization and different forms of intermittent fasting represent important components of the nutritional strategy in metabolic syndrome, with effects on weight control as well as inflammation and insulin sensitivity [87]. An intensively studied model is 16:8 time-restricted feeding (TRF), in which energy intake is compressed into an 8 h window, while only water is consumed during the remaining 16 h. This pattern belongs to intermittent-fasting approaches that target both energy balance and the circadian rhythms of glucose and lipid metabolism. In a preclinical experiment in mice fed a normal diet, 12 weeks of a 16:8 protocol led to an approximately 15% reduction in body weight at the end of the period, a significant difference compared with the group with ad libitum food access. In the same model, TRF markedly improved glucose tolerance and insulin sensitivity, shown by a decrease in the area under the curve during the glucose tolerance test and a more rapid decrease in glycemia during the insulin sensitivity test. Measurements of insulin secretion after glucose loading showed a faster and higher-amplitude response of pancreatic beta cells at 2.5, 5, and 15 min, suggesting positive regulation of insulin secretion induced by TRF. In addition, in these mice with low baseline inflammation, TRF significantly decreased serum IL-6 levels, while IL-1β and TNF-α remained unchanged, indicating a modest anti-inflammatory effect in a physiological context [1,108,109]. Exploration of the same 16:8 protocol in mice fed a high-fat diet, used as a model of obesity and Western diet, showed more pronounced effects. TRF reduced body weight, significantly improved glucose tolerance and insulin resistance, and attenuated low-grade inflammation, at the same time improving hepatic steatosis and reducing adipocyte diameter in epididymal white adipose tissue. These changes support the role of intermittent fasting in weight control and adipose-tissue remodeling in the context of metabolic syndrome, although the data come from animal models. In the same model, TRF increased thermogenesis in brown adipose tissue (BAT) after cold exposure, associated with increased gene and protein expression of UCP1 and PGC-1alpha, providing a plausible mechanism for increased energy expenditure and reduction in fat stores. TRF proved to reduce the expression of hepatic genes involved in lipogenesis and may therefore benefit patients with non-alcoholic fatty liver disease, although these findings require further clarification. Other preclinical studies suggest that the favorable effects of TRF on insulin resistance may involve activation of PPAR and SIRT3 pathways and remodeling of the gut microbiota, including browning of white adipose tissue and VEGF-mediated thermogenesis and alternative macrophage activation, but these mechanisms were not directly investigated in the described 16:8 protocol [110]. From the perspective of systemic inflammation and the prothrombotic status associated with metabolic syndrome, these effects of TRF on IL-6, hepatic steatosis, and adipose mass overlap with the general need for interventions that simultaneously target metabolic control and inflammation reduction [6,111]. However, the results on BAT thermogenesis activation, reduction in inflammatory markers, and improvement of metabolic syndrome were obtained in animals and have not yet been convincingly demonstrated in humans for the 16:8 model, which requires caution in direct extrapolation to clinical practice [111]. In parallel with meal-timing interventions, programs focused on dietary fiber and prebiotics support weight control and metabolic parameters through microbiota-mediated mechanisms. For example, a diet rich in fermentable fiber in patients with type 2 diabetes mellitus improved glycemic control and weight loss, associated with selective promotion of approximately 15 SCFA-producing strains and increases in butyrate and acetate, with favorable effects on GLP-1 secretion, insulin sensitivity, and lipid metabolism. Other clinical studies with inulin or inulin-type fructans in overweight individuals or those with metabolic syndrome showed greater weight loss than placebo, reductions in diastolic blood pressure, and improvement in insulin resistance, together with increased *Bifidobacterium* and decreased *Desulfovibrio*, microbial changes correlated with anthropometric and metabolic improvements [93]. Overall, the available evidence indicates that interventions targeting meal timing, especially TRF, can modulate body weight, insulin resistance, inflammation, and adipose-tissue distribution in animal models, whereas fiber- and prebiotic-rich interventions support weight control and optimization of metabolic response through the gut–microbiota axis in humans [6,93,111].

Integrating these strategies with the previously described plant-based dietary patterns may provide a complex approach focused on both diet quality and the temporal structure of energy intake. Recent studies also revealed that diet microbiota modifications and gut-barrier repair reduce endotoxin translocation and levels of cytokines such as IL-6, suggesting a mechanism through which fiber and prebiotics may contribute to reducing systemic inflammation associated with chronic inflammatory diseases [110,112].

## 6. Conclusions

Integrated nutritional strategies for managing metabolic syndrome should prioritize the adoption of a dietary model centered on whole plant foods, vegetables, fruits, whole grains, and healthy fats, while limiting processed foods. Complementary approaches, such as time-restricted eating and the integration of prebiotics or probiotics, may amplify metabolic and anti-inflammatory benefits. Evaluating the efficacy of these interventions requires the use of multiple biomarkers, including inflammatory, metabolic, and oxidative-stress indicators, measured by standardized laboratory methods. Anti-inflammatory diets can help manage cardiometabolic disorders, but they face key limitations: inconsistent definitions, lack of self-reported data, and variable individual responses depending on intestinal permeability. Moreover, while the Mediterranean diet lowers inflammation markers such as CRP and IL-6, thus improving blood pressure and lipid and amino acid profiles, complex lifestyle changes are required for long-term beneficial effects. Overall, nutritional orientation toward an anti-inflammatory profile represents a fundamental and applicable component for the prevention and improvement of metabolic syndrome and its associated cardiovascular complications. Future research integrating metabolomics, genomics, and artificial intelligence could improve personalized cardiometabolic management, moving towards individualized precision medicine.

## Figures and Tables

**Table 4:** Impact of pro- and anti-inflammatory diets on biochemical and cellular parameters.

Biochemical/Cellular Parameter	Conventional Omnivore Model (Western Diet)	Anti-Inflammatory Vegan/Mediterranean Model	Pathophysiological Significance and Cardiometabolic Impact	References
Insulin signaling pathway	Inhibited via JNK- and IKK-mediated serine phosphorylation of IRS-1.	Active, with normal tyrosine phosphorylation of IRS-1 and AKT activation.	Determines the rate of GLUT-4 translocation and susceptibility to type 2 diabetes.	[13,16,77]
Adipokine profile	Elevated leptin (due to resistance), decreased adiponectin.	Normalized leptin, significantly increased adiponectin.	Adiponectin exerts direct anti-inflammatory and insulin-sensitizing effects	[18,19,30]
Adipose immune infiltration	Dominated by M1 macrophages (>12%), CD8+ and Th1 lymphocytes.	Dominated by M2 macrophages, Treg (~50%) and Th2 lymphocytes.	Governs local inflammatory status and systemic cytokine secretion.	[21,78]
Acute-phase proteins	hsCRP, fibrinogen, serum Amyloid A persistently elevated.	hsCRP and acute-phase proteins at minimal, physiological levels.	Indicates the degree of hepatic inflammatory activation and blood viscosity.	[22,31,79]
Coagulation homeostasis	Elevated PAI-1, decreased t-PA activity, increased D-dimers.	Optimized PAI-1/t-PA ratio, decreased fibrinogen, non-thrombogenic status.	Defines the risk of arterial thrombosis and venous thromboembolism.	[3,80]
Endothelial dysfunction	Uncoupled eNOS, decreased NO, high expression of ICAM-1/VCAM-1.	Actively coupled eNOS, elevated NO, minimal expression of adhesion molecules.	Controls vasodilation capacity and atheromatous plaque initiation.	[26,55]

**Abbreviations:** AKT, Protein kinase B; CD8+, Cluster of differentiation 8-Positive; eNOS, Endothelial nitric oxide synthase; GLUT4, Glucose transporter 4; hsCRP, High-sensitivity C-reactive protein; ICAM-1, Intercellular adhesion molecule-1; IKK, IκB kinase; IRS-1, Insulin receptor substrate 1; JNK, c-Jun N-terminal Kinase; NO, Nitric oxide; PAI-1, plasminogen activator inhibitor-1; Th1, T helper 1, Th2, T helper 2, t-PA, Tissue-type plasminogen activator; Treg, Regulatory T cells; VCAM-1, Vascular cell adhesion molecule-1.

**Table 5:** Cardiometabolic effects of microbial metabolites on key molecular targets.

Microbial Metabolite	Primary Dietary Source	Bacterial Biosynthetic Pathway	Key Molecular Targets	Cardiometabolic and Pathophysiological Effect	References
TMAO	Red meat, eggs, fish (choline, carnitin, lecithin)	Conversion to TMA by colonic microbiota, followed by FMO3-mediated hepatic oxidation	NF-κB, NLRP3 inflammasome, SIRT1, SIRT3, NADPH oxidase	Pro-inflammatory endotheliitis, mitochondrial oxidative stress (decreased SOD2), platelet hyperreactivity, accelerated atherogenesis	[94]
PAGIn	Protein-rich diets (phenylalanine)	Phenylalanine catabolism by microbiota and host conjugation	α2A-adrenergic receptors	Increased platelet reactivity, elevated risk of arterial thrombosis	[95]
(SCFAs)	Soluble fiber, complex carbohydrates	Bacterial fermentation of polysaccharides	GPR41, GPR43, Histone Deacetylases (HDAC)	Systemic anti-inflammatory effects, lower blood pressure, improved lipid profile, Treg stabilization	[96,97,98]
Indoles (IPA)	Dietary tryptophan	Bacterial tryptophanases	AhR receptor, NAD metabolism	Antioxidant, protection against diastolic dysfunction, maintenance of endothelial barrier integrity	[87,88]
Secondary bile acids	Primary bile acids	Bacterial deconjugation and dehydroxylation	Nuclear FXR, PXR, VDR; membrane TGR5 receptor	Modulation of lipid and glucose metabolism, NO-dependent vasorelaxation; excess deoxycholic acid induces cytotoxicity	[90,99]

**Abbreviations**: AhR, Aryl hydrocarbon receptor; FMO3, Flavin-containing monooxygenase 3; FXR, Farnesoid X receptor; GPR41, G protein-coupled receptor 41; GPR43, G protein-coupled receptor 43; HDAC, Histone deacetylase; IPA, Indole-3-propionate; NADPH, Nicotinamide adenine dinucleotide phosphate; NF-κB, Nuclear factor-kappa B; NLRP3, NOD-, LRR- and pyrin domain-containing protein 3; NO, Nitric oxide; PAGln, Phenylacetylglutamine; PXR, Pregnane X receptor; SCFAs, Short-chain fatty acids; SIRT1, Sirtuin 1; SIRT3, Sirtuin 3; SOD2, Superoxide dismutase 2; TGR5, Takeda G protein-coupled receptor 5; TMA, Trimethylamine; TMAO, Trimethylamine N-oxide; Treg, Regulatory T cell; VDR, Vitamin D receptor.

**Table 6:** Effects of dietary models on endothelial function.

Dietary Model	Bioactive Nutritional Characteristics	Impact on Flow-Mediated Dilation (FMD)	Estimated Cardiovascular Risk Reduction	Cellular and Molecular Mechanisms	References
DASH diet	Rich in potassium, magnesium, calcium, fiber; low in sodium and saturated fats.	2.5% increase at 6 months; 3.1% at 18 months.	20–32% reduction (at 6 months); 25–40% (at 18 months).	Reduction in oxidative stress, increased NO bioavailability, optimization of the Na^+^/K^+^-ATPase pump.	[83,104]
Mediterranean diet (“Green”)	Fortified with polyphenols (Mankai, green tea), monounsaturated fatty acids (oleic acid).	Significant improvement in arterial compliance.	Consistent improvement in lipid profile and blood pressure.	Prevention of eNOS uncoupling, direct free-radical scavenging, NF-κB inhibition.	[50,55]
Plant-based diet (Counseling)	Exclusion of animal fats, massive intake of plant sterols and soluble fiber.	Significant FMD improvement within 4 months; decreased PWV.	7.6% reduction in total cholesterol and 13.6% reduction in LDL.	Reduction in arterial stiffness, stimulation of NO synthesis, decreased circulating LDL.	[55,105]
Nordic diet	Fatty fish (omega-3), berries, whole grains (oats, rye), rapeseed oil.	Amelioration of microvascular vasoreactivity.	hsCRP reduction and stabilization of blood pressure.	Antioxidant action of anthocyanins, stimulation of colonic SCFA synthesis (β-glucan).	[106]
Traditional Japanese (Washhoku)	Fish rich in omega-3, soy foods, regular green tea consumption.	Marked improvement in brachial artery dilatation.	Direct antioxidant protection of endothelial cells.	eNOS activation via PI3K/Akt signaling pathways mediated by catechins (EGCG).	[107]

**Abbreviations**: EGCG, Epigallocatechin gallate; eNOS, Endothelial nitric oxide synthase; FMD, Flow-mediated dilation; hsCRP, High-sensitivity C-reactive protein; LDL, Low-density lipoprotein; NF-κB, Nuclear factor-kappa B; NO, Nitric oxide; PI3K, Phosphoinositide 3-kinase; PWV, Pulse wave velocity; SCFA, Short-chain fatty acid.

## Data Availability

The data presented in this study are available in this article.
